# Effects of different high pressure steaming processes on sensory properties, water-binding capacity, antioxidant and aroma compounds of *Ipomoea batatas* (L.) Lam

**DOI:** 10.3389/fnut.2025.1597754

**Published:** 2025-05-29

**Authors:** Anqi Cao, Mingyi Yang, Chaofan Hu, Asem M. Abdelshafy, Jicheng Bao, Sining Yan, Guoquan Lu, Yuge Guan, Jiyu Cheng, Linjiang Pang, Xinghua Lu

**Affiliations:** ^1^Jiyang College of Zhejiang A&F University, Zhuji, China; ^2^College of Food and Health, Zhejiang A&F University, Hangzhou, China; ^3^Institute of Food Science, Wenzhou Academy of Agricultural Science, Wenzhou, China; ^4^Department of Food Science and Technology, Faculty of Agriculture, Al-Azhar University - Assiut Branch, Assiut, Egypt

**Keywords:** Sweet potato, high pressure steaming, sensory properties, water status, aroma, correlation analysis

## Abstract

**Background:**

High pressure steaming is a convenient and household cooking method to heat the food evenly and retain original nutrition. This study explored the effects of high pressure steaming processes on the sensory properties, nutritional value, phenolic compounds, water status, and volatile compounds of sweet potato “Xinxiang” and “Pushu32.”

**Results:**

Compared with soft and exquisite “Pushu32”, chewy “Xinxiang” possessed a significantly lower fiber score and exhibited greater changes in adhesiveness, cohesiveness, chewiness, and gumminess after steaming. Sensory evaluation revealed the optimal steaming conditions were 125°C (232.1 kPa) × 15 min for “Xinxiang”, and 115°C (169.1 kPa) × 25 min for “Pushu32”. Low-field nuclear magnetic resonance uncovered that more than 90% of water in sweet potato was immobilized water, and 125°C steaming enhanced bound water proportion, which might contribute to the adhesive texture. The treatment at 125°C for 15 min showed higher anthocyanin, soluble sugar contents, and lower ascorbic acid content in sweet potato. Moreover, 75 volatile components were identified in steamed sweet potato using GC–MS. Correlation analysis implied that benzeneacetaldehyde, trans-*β*-ionone, *α*-gurjunene, and nonanal were pivotal aroma compounds of steamed sweet potato. The trans-*β*-ionone, nonanal, and decanal might be correlated with the dull color of sweet potato. The presence of polyphenols might be conducive to *α*-gurjunene production and wood aroma.

**Conclusion:**

The present study offered the optimal high pressure steaming process for sweet potato and provided new insights into the sensory quality formation and personalized control.

## 1 Introduction

Sweet potato (*Ipomoea batatas* L.) is an important economical crop in the world for food security addressing and poverty alleviation. Sweet potatoes are known as “longevity food,” which are a good natural source of carbohydrates and energy, rich in polyphenols (0.32–13.82 μg·g^−1^), carotenoids (0.22–559.70 μg·g^−1^), dietary fiber (0.54–3.52%), vitamin C (8.17–66.09 mg·100 g^−1^) and various minerals (1, 2). Due to the characteristics of high output and strong vitality, sweet potatoes are widely cultivated in more than 120 countries and regions in the world (3). In recent years, with people’s pursuit of a healthy diet and nutrition balance, processed sweet potato snacks and products are becoming favored by popularity.

Sweet potatoes’ taste and flavor result from different kinds of cooking methods, including steaming, boiling, roasting, and frying, which directly affect consumers’ acceptance and market competitiveness (4). Roasting and frying are processed at higher temperatures, usually produce special flavors through the Maillard reaction, but are prone to loss nutrition and generate risk substances (4). Boiling with less oil is healthy, but is prone to lose water soluble nutrients (4). Steaming, as a traditional and healthy cooking method, well maintains the flavor and nutritional contents of food ingredients (5). After 100°C steaming for 40 min, the total starch content of purple sweet potato decreased, while the soluble sugar content increased with no significant differences in anthocyanin content in most sweet potato varieties (6). The migration and distribution of water in the steaming process directly determines the final taste characteristics of food, and affects the sensory properties, including texture, taste and aroma (7). The water vapor and heat during steaming increased the proportion of bound water, thus enhancing the water-starch, water-gluten interactions, and improving the texture (8). However, the effects of the steaming conditions (temperature, time, pressure, etc.) on the moisture state in food, which affected the food taste were still less investigated.

The high pressure steaming process enabled the rapid release of moisture inside the food, and promoted uniform temperature distribution, resulting in improving food softening, and increasing the release and transformation of volatile components. For example, steaming induced the non-enzymatic degradation of unsaturated fatty acids in green tea and facilitated the accumulation of key volatile compounds (9). At the beginning of the steaming process, a large number of aroma compounds were produced and released by the potato matrix. With the increase of heating time, the starch particles were gradually destroyed, and the produced starch macromolecular chains interacted with the aromatic compounds (10). “Xinxiang” and “Pushu32” were 2 popular sweet potato varieties, suitable for steaming process. “Xinxiang” with yellow root flesh was famous for a fragrance of chestnut, while “Pushu32” had high content of carotene, thus possessed a red flesh. However, the correlation between the steaming process and volatile components of “Xinxiang” and “Pushu32” sweet potatoes is unclear.

Therefore, this study investigated the effects of the high pressure steaming process on the sensory properties of two widely used steaming sweet potato varieties, “Xinxiang” and “Pushu32”, including color, texture, taste, and aroma. Different thermal temperatures, pressures and times were conducted to figure out the optimal processing method. The sensory evaluation was combined with objective detection techniques such as texture analyzer, gas chromatography–mass spectrometry (GC–MS), and low-field nuclear magnetic resonance (LF-NMR). The key aroma substances were characterized by GC–MS. The steaming parameters (time, temperature, and pressure) were correlated to construct a comprehensive evaluation system for the quality of steamed sweet potatoes, breaking through the limitations of traditional single-index research. Besides, the mechanism of processing technologies on water dynamics, texture, taste, and bioactive compounds content was explored to provide a new perspective for process optimization and sweet potato product development.

## 2 Materials and methods

### 2.1 Materials and experimental design

Sweet potato *Ipomoea batatas* (L.) Lam. cv. “Xinxiang” and “Pushu32” were collected from our planting base (119.7° E, 30.3° N, Hangzhou, China) in January, 2024. Fresh sweet potato with uniform size, and no mechanical injury were selected and washed for further use. Sweet potato were cut into chunks (diameter 5 cm and height 2 cm) with a knife along the equator, followed by vacuum packing using high-temperature resistant cooking bags. Each bag was a replicate and 15 replicates were performed in each group. According to our preliminary experiment, the sweet potato chunks were cooked in a high-pressure steam sterilizer (GI54DS, Zealway Instrument Inc., USA) under conditions of “105°C (120.8 kPa), 35 min,” “115°C (169.1 kPa), 25 min” and “125°C (232.1 kPa), 15 min.” After cooling to room temperature, the samples were taken for determination of color, texture, sensory evaluation, and volatile compounds. The remaining sweet potato chunks were cut into small pieces, frozen in liquid nitrogen, and freeze-dried for further use. In this study, chemicals used for volatile compounds analysis were chromatographical pure, while the other chemical substances were analytical pure (Macklin Biochemical Technology Co., Ltd., Shanghai, China).

### 2.2 Quality indexes of steamed sweet potato

The color of sweet potato chunks was measured by colorimeter (CR-10, CHNSpec Co., Ltd., Hangzhou, China) under L*a*b* model according to the published method (11). L* ranged from 0 to 100 represents lightness from black to white; a* from negative to positive represents greenness to redness; b* from negative to positive represents blueness to yellowness. Data were collected from the opposite sides on the center surface of sweet potato chunks with 3 replicates for each experiment.

The sample texture was detected from the center of sweet potato chunks through a texture analyzer (TMS-PRO, Food Technology Corporation, Virginia, USA) based on the reported method (12). A P/5 cylindrical probe was used. Pre-test speed = 1.5 mm s^−1^; test speed = 1 mm s^−1^; post-test speed = 1.5 mm s^−1^. The compression ratio was 60%, and the puncture distance was 40.0 mm. After the sample is subjected to pressure deformation, if the surface of the sample is sticky, a negative force will be generated. In food, it can be interpreted as adhesiveness, which is the negative force-receiving area of the first compression. Cohesiveness is defined as the ratio of the normal force-bearing area of the first compression to that of the second compression, and reflects the tensile strength of the food. Samples with good cohesiveness are also easier to keep the probe/teeth clean. Springiness is the height to which food can recover between the end of the first force and the beginning of the second force. Gumminess is defined as firmness (the maximum force value during the first compression) × cohesiveness, and used to describe the taste of semi-solid food. Chewiness is defined as stickiness × elasticity, and can be interpreted as the energy required to chew solid food. Adhesiveness, cohesiveness, springiness, gumminess, and chewiness were determined with 3 replicates for each experiment.

### 2.3 Sensory evaluation

The steamed sweet potato chunks were cooled to room temperature and randomly placed for sensory evaluation. Teachers and students who complete the sensory evaluation course were selected to be sensory evaluators. The sensory evaluation was conducted according to American Society for Testing and Materials (ASTM) International (13). Samples were scored by 10 male and 10 female sensory evaluators aged 20–30, based on sweetness, aroma, texture and comprehensiveness. During the evaluation, each person scored independently and had no contact with each other. A 20-point scale of scoring method was used for sensory evaluation. Sweetness from none to high ranged 0–20; aroma with sweet potato fragrance from none to strong ranged 0–20; color with no brown/black spots and presented uniform light yellow was scored 20; texture with the dense, soft, and no-fiber was scored 20; and the comprehensiveness was based on the overall condition of sweet potato chunks, ranged 0–20. Detailed sensory evaluation criteria are shown in Table 1.

**Table 1:** Sensory evaluation criteria of steamed sweet potato.

Sensory standard	0–5	5–10	10–15	15–20
Sweetness	Slightly sweet	Moderate	Relatively high	High
Aroma	Faint	Slight	Moderate	Strong
Color	Poor with a black heart and many black spots	Pale yellow with brown spots on the surface	Pale yellow with a dull and relatively consistent color	Pale yellow in color with a good and uniform hue
Fiber	Intense fibrous sensation and hard to chew	A certain degree of fibrous sensation with dense meat	Little fibrous sensation and fine without tendons	No fibrous texture and softy
Overall	Poor	Average	Relatively good	Delicious

### 2.4 Low-field nuclear magnetic resonance

The water status of fresh and steamed sweet potato samples were measured by low-field nuclear magnetic resonance (MesoMR23-060 V-I, Niumag Co., Ltd., Suzhou, China) according to published method (14). The sweet potato chunks were pulverized and put into a 20 mm diameter cylindrical glass tube. The Carr-Purcell-Meiboom-Gill method was applied to conduct the relaxometry analysis at 32°C. Magnetic field strength = 0.5 T; spectrometer frequency = 21 MHz; number of scans = 4; number of echoes = 7,500; time echo = 0.2 ms; and time waiting = 1,500 ms. Data analysis was performed using Niumag Multi-Exp Inv Analysis software. Each experiment was carried out in triplicate.

### 2.5 Determination of total polyphenol, flavonoid, and anthocyanin contents

As described in the former study (15), 0.5 g samples were added into 20 mL pre-cooled 1% HCl-methanol solution and mixed uniformly. The mixture was extracted at 4°C for 20 min in the dark. The supernatant was the extraction for measurement of absorbance value at 280 nm, 325 nm, 530 nm, and 600 nm. Standard calibration curves of gallic acid, rutin, and cyanidin were drawn for quantification of total polyphenol at 280 nm, flavonoid at 325 nm and anthocyanin (the difference between the absorbance values at 530 nm and 600 nm), respectively. Each experiment was carried out in triplicate.

### 2.6 Determination of ascorbic acid content

The ascorbic acid content was measured using an assay kit (BC1230, Beijing Solarbio Science & Technology Co., Ltd., China) following the instruction with 3 replicates for each experiment.

### 2.7 Determination of total soluble sugar content

The total soluble sugar content was determined using an assay kit (BC0030, Beijing Solarbio Science & Technology Co., Ltd., China) following the instruction with 3 replicates for each experiment.

### 2.8 Volatile compounds analysis

Gas chromatography–Mass spectrometry (GC–MS) pretreatment was carried out according to a reported protocol (16). The steamed sweet potatoes were cooled to room temperature. 1.0 g sample was placed in a headspace bottle, heated to 60°C, and shaken for 5 min. The volatile components were trapped by the SPME fiber and desorbed in the GC injector port at 250°C for 3 min. The detection was performed on the GC System (Thermo Scientific™ TRACE™ 1310, USA), coupled with an ISQ mass spectrometer (Thermo Fisher Scientific, USA) and an HP-5 MS elastic quartz capillary column (30 m × 0.25 mm i.d. × 0.25 μm). The high-purity helium carrier gas flow rate was 1.2 mL min^−1^. The running program was set as follows: the initial 35°C was kept for 2 min, followed by a ramping period from initial 35°C to 180°C at a rate of 8°C min^−1^ and kept for 2 min, and finally increased to 280°C at a rate of 15°C min^−1^, and held for 1 min. The temperatures of the inlet, MS transfer line, and ion source were 250°C, 280°C, and 300°C, respectively. The electron impact (EI) ionization voltage was 70 eV. Full scan collection mode was adopted, with m/z ranging from 35 to 400. Aroma components were tentatively identified using the National Institute of Standards and Technology (NIST17) Library and quantified by comparing peak areas with the internal standard of 2-octanol.

### 2.9 Correlation analysis

Spearman correlation analysis for association analysis between quality indexes and main aroma components of sweet potato was conducted according to a reported method (17).

### 2.10 Statistical analysis

All data were presented in mean ± standard deviation and analyzed using one-way analysis of variance (SPSS V27.0 software, IBM Inc., Chicago, USA). Significant differences between groups were determined through Duncan’s multiple difference analysis (*p <* 0.05). Graphs were produced through Origin 2021 and GraphPad Prism10 software.

## 3 Results and discussion

### 3.1 Color characteristics of steamed sweet potato

**Figure 1:**
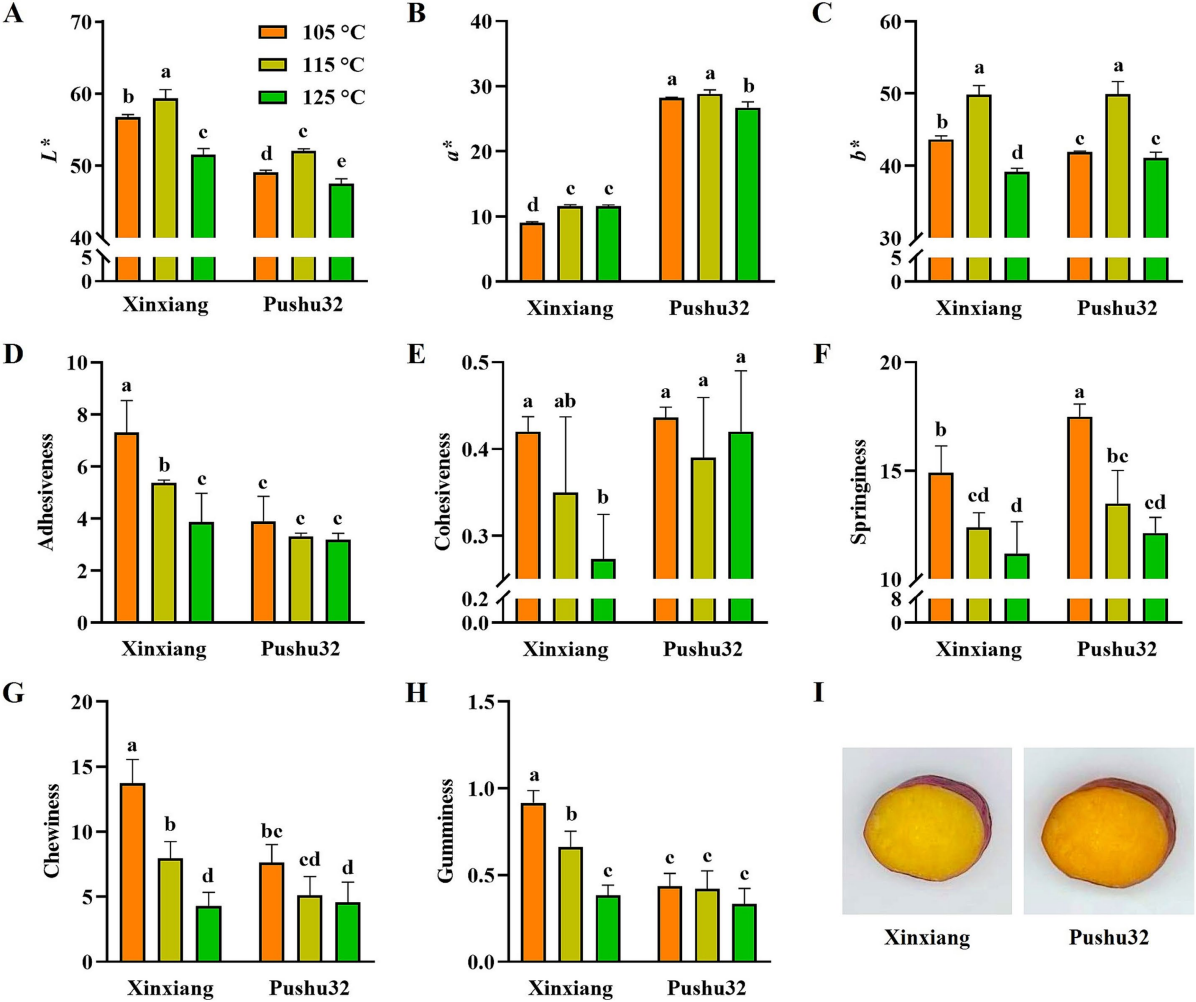
Color and texture characteristics of steamed sweet potato “Xinxiang” and “Pushu32” at different temperatures **(A)** L*, **(B)** a*, **(C)** b*, **(D)** Adhesiveness, **(E)** Cohesiveness, **(F)** Springiness, **(G)** Chewiness, **(H)** Gumminess and **(I)** appearance of steamed sweet potato “Xinxiang” and “Pushu32” at different temperatures. Different letters represent significant differences among groups using duncan’s multiple-range test (*p* < 0.05).

Color characteristics of L*, a*, b* of steamed sweet potato “Xinxiang” and “Pushu32” under different temperatures were determined (Figures 1A–C). Consistent with the appearance of sweet potato, “Pushu32” was evidently redder, thus exhibiting more than doubled a* value of “Xinxiang” (Figure 1B). Compared with other steaming processes at 105°C and 125°C, “115°C (169.1 kPa), 25 min” steamed sweet potato possessed significantly higher L* and b* (*p* < 0.05), showing lighter and yellower color (Figures 1A,C). In comparison, no obvious difference in a* value was found between different steaming processes (Figure 1B). It was reported that the steamed sweet potato became redder and yellower after steaming (18). However, 25 and 35 min steaming had no significant effects on L*, a* and b* values of purple sweet potato (19), indicating that steaming duration made no difference in color changes. Thus, we speculate that an increase in temperature might promote the release of carotenoids and anthocyanins, while an excessively high temperature might lead to pigment degradation.

### 3.2 Texture characteristics of steamed sweet potato

It was reported that the texture profile analysis (TPA) is a method to mimic the food sensor in the mouth and measure the characteristics of the food texture without bias (20). Therefore, to investigate the effects of different steaming processes on texture properties of sweet potato “Xinxiang” and “Pushu32”, texture profile analysis, including adhesiveness, cohesiveness, springiness, chewiness, and gumminess were comprehensively detected (Figures 1D–H). For “Xinxiang”, all texture indexes presented a decreasing trend with the increase of temperature and pressure (Figures 1D–H). Similarly, the springiness and chewiness of “Pushu32” were significantly lower after 125°C (232.1 kPa) steaming than that after 105°C (120.8 kPa) steaming (Figures 1F,G). Moreover, during the process of sweet potato from raw to cooked, the firmness reduced with the steaming time duration and remained similar after 15 min (21). In agreement with our results, the hardness, gumminess, chewiness, and resilience of sweet potato reduced with thermal pressure increase, which could reach a higher temperature (20). Besides, the gumminess, chewiness and resilience of sweet potato decreased with pressure cooking (20). An increase in temperature might accelerate the starch pasting, resulting in a softer texture of sweet potato (6). In addition, the amylose content, amylopectin structure of sweet potato could contribute to the firmness and viscosity sensory differences of sweet potatoes (22). Furthermore, the changes in adhesiveness, cohesiveness, chewiness, and gumminess of “Xinxiang” under different steaming temperatures were greater than that of “Pushu32”. This might be related to the buffering effects of substances such as cellulose, pectin and sugar in different varieties of sweet potato (23).

### 3.3 Sensory characteristics of steamed sweet potato

**Figure 2:**
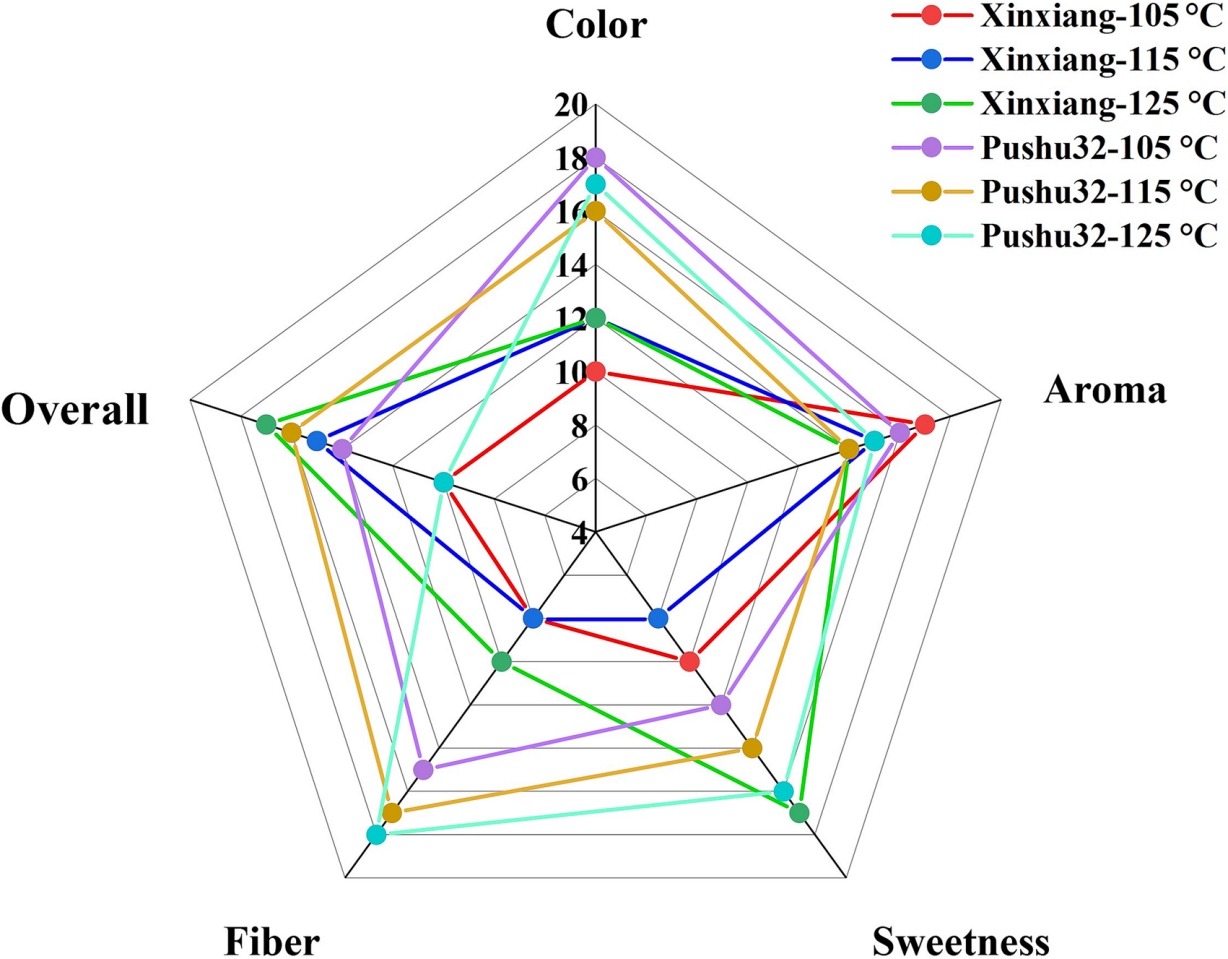
Sensory profiles of steamed sweet potato “Xinxiang” and “Pushu32” at different temperatures.

Sensory properties of steamed sweet potato were determined to evaluate the influence of different steaming processes on flavor. Figure 2 shows that the top three overall ratings in sensory evaluation were Xinxiang 115°C, Pushu32 115°C, and Xinxiang 125°C. Sweet potato with high satiety is rich in dietary fiber, and helps promote intestinal movement and purgation (24). Compared to “Pushu32” with soft and exquisite texture, significantly lower fiber scores were found in “Xinxiang” (Figure 2), indicating the chewier texture of “Xinxiang”. Similarly, the sticker texture of starchy food was obtained after steaming (25). It was reported that steamed root vegetables usually obtained a softer sensory due to the cell shrinkage, and the loss of cell turgor and adhesion (26). In addition, thermal treatment might be accompanied by the degradation of starch and the improvement of soluble solids, which was conducive to the saccharification of sweet potato (27). Among three heating temperatures, the sweet potato of both varieties processed at 125°C had the highest sweetness (Figure 2). In comparison, the sweetness of “Xinxiang” was lower than that of “Pushu32” at 105°C and 115°C, but both peaked at 125°C (Figure 2). Obviously, “Pushu32” with red light color was more favorable by consumers, presenting a one-third higher color score than “Xinxiang” (Figure 2). However, no significant effect was shown on sweet potato color under different temperatures (*p* > 0.05). Above all, in terms of sensory characteristics, the optimal steaming process was 125°C (232.1 kPa) × 15 min for “Xinxiang,” and 115°C (169.1 kPa) × 25 min for “Pushu32”.

### 3.4 Water status of steamed sweet potato

Water state is closely related to the edible quality of sweet potato (28). Three water populations, including bound water, immobilized water, and free water were detected using low-field nuclear magnetic resonance. Low-field nuclear magnetic resonance measures the degree of free water according to the varying relaxation time T_2_, which was determined by the chemical environment where protons reside (29). The shorter relaxation time T_2_ is, the more tightly water is bound to the material, and the less easily it flows (30). Therefore, the relaxation time T_2_ can be used to understand the migration rule of water in the steaming process of sweet potato and uncover the phase characteristics of water.

**Figure 3:**
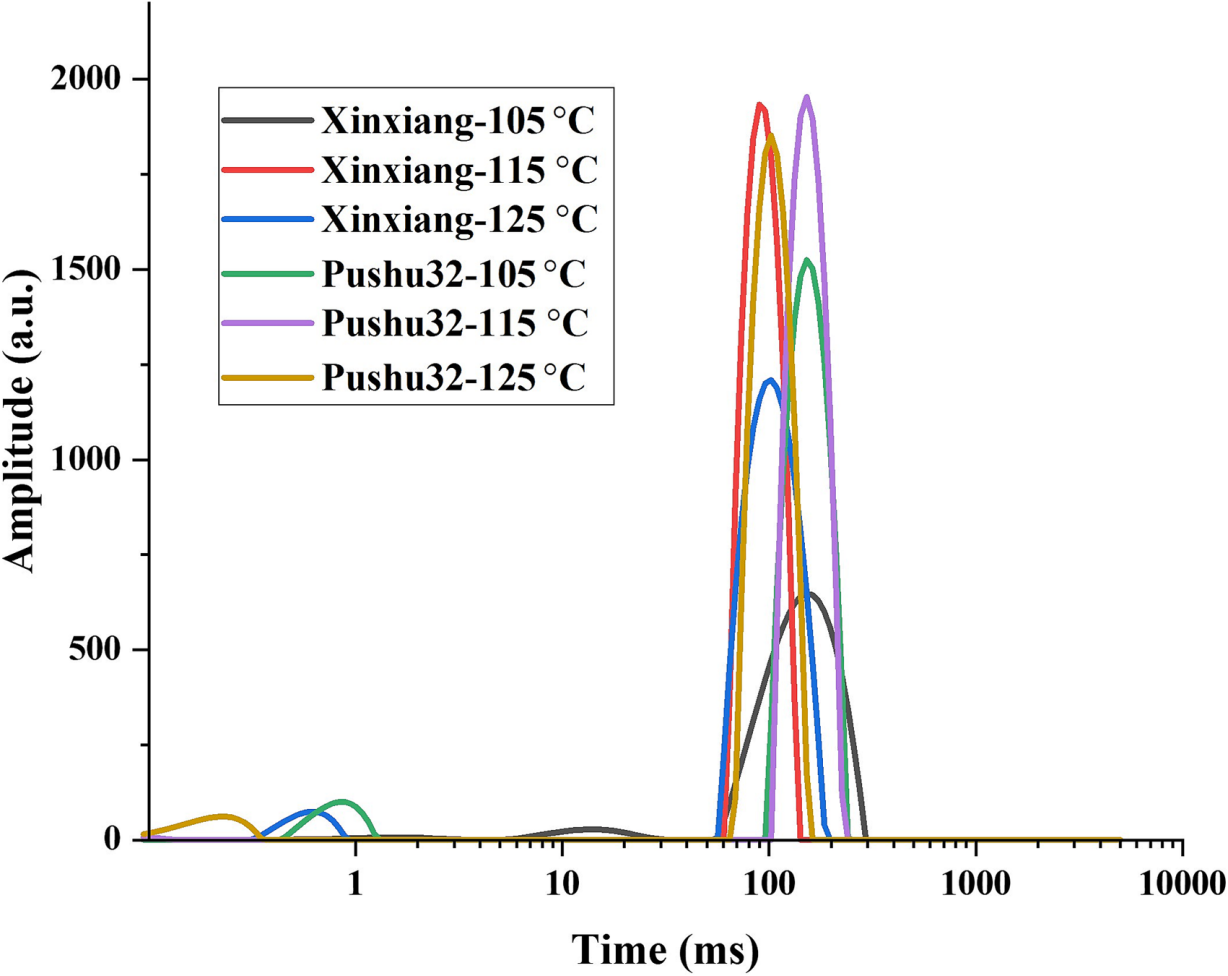
Water status of steamed sweet potato “Xinxiang” and “Pushu32” at different temperatures.

Bound water and immobilized water were detected in this study (from left to right in Figure 3, respectively), and the signal of immobilized water reached more than 90% of the whole signal value. Results demonstrated that with the rise of temperature and pressure, the content of immobilized water increased, accompanied by the reduced relaxation time T_2_ (Figure 3), indicating reduced water mobility. Among all treatments, steamed sweet potatoes at 125°C obtained the highest bound water content in both “Xinxiang” and “Pushu32” (Figure 3). Obviously, high pressure steaming introduced water vapor to increase the content of immobilized water. In agreement with our results, compared with raw samples, roasted sweet potato had a higher proportion of free water and a lower proportion of bound water, causing an increased internal free water gradient than the external, thus promoting the water diffusion from inside to outside and softening the texture (31). It was reported that steaming could reduce the mobility of hydrogen protons, which contributed to the interaction between water and starch, thus enhancing the proportion of bound water to a certain extent (8). Additionally, more than 85% of water existent status of Chinese steamed buns was immobilized and free water, which significantly correlated with the dynamic changes of hardness and adhesiveness during cooling (*p* < 0.01) (32).

### 3.5 Antioxidants and soluble sugar contents of steamed sweet potato

**Figure 4:**
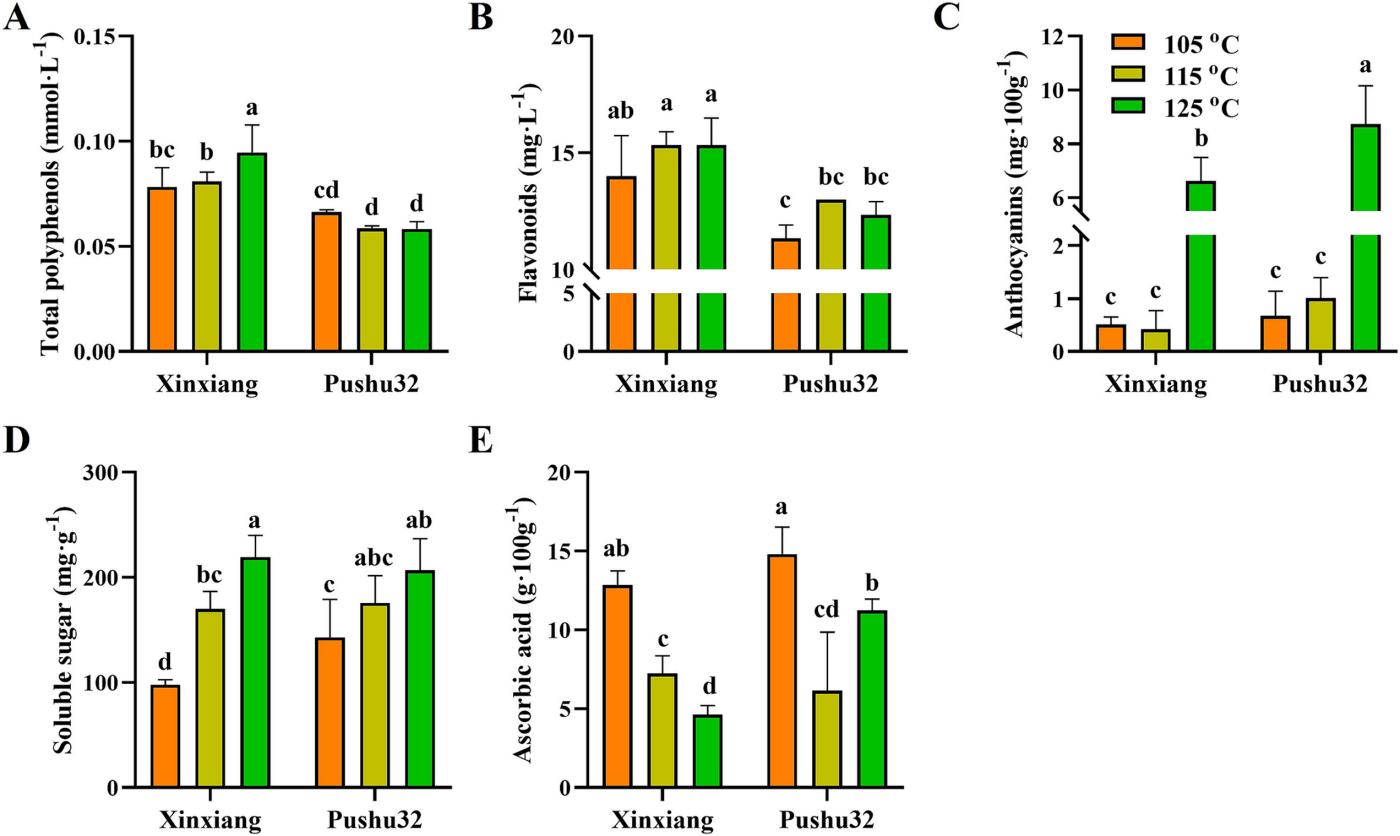
Antioxidants and soluble sugar contents of steamed sweet potato “Xinxiang” and “Pushu32” at different temperatures Contents of **(A)** total polyphenols, **(B)** flavonoids, **(C)** anthocyanins, **(D)** soluble sugar, and **(E)** ascorbic acid of steamed sweet potato “Xinxiang” and “Pushu32” at different temperatures. Different letters represent significant differences among groups using duncan’s multiple-range test (*p* < 0.05).

Steaming was a better method to obtain 9.44% higher polyphenol content and 81.40% higher antioxidant activities of sweet potato leaves, compared with boiling, microwave, baking and frying cooking methods (33). To investigate the effects of different steaming processes on nutritional values of sweet potato “Xinxiang” and “Pushu32”, contents of total polyphenols, flavonoids, anthocyanins, soluble sugar, and ascorbic acid were determined (Figure 4). In comparison, “Xinxiang” possessed significantly higher polyphenol and flavonoid contents than “Pushu32” (*p* < 0.05, Figures 4A,B). Generally, the polyphenol stability decreases with the increase of heating temperature, pressure and the extension of heating time (34). Despite the increase in steaming temperature, the shorter steaming time, especially “125°C, 15 min” well maintained a significantly higher content of anthocyanins in sweet potato than other processes (*p* < 0.05, Figure 4C). In addition, steaming had no effect on the total phenolic content of asparagus, and the total antioxidant activity of wild violet asparagus increased by 46% after steaming (35).

Cooking facilitated starch gelatinization and inactivated amylase inhibitors, hence enhancing starch digestibility (25). Consistently, our results showed that the soluble sugar content of both varieties of sweet potato gradually improved with the increase of temperature (Figure 4D). Besides, cooking (steaming, boiling, or baking) of orange-fleshed sweet potato was accompanied with decreased starch content from 18.15 to 7% and more than tripled soluble sugar (36).

Ascorbic acid was sensitive to high temperatures, thus its content decreased after thermal processing (37). In accordance with the former research, the content of ascorbic acid in “Xinxiang” sweet potato decreased with the increase of temperature after steaming (Figure 4E). In particular, the contents of ascorbic acid in “Pushu32” sweet potatoes from high to low were under 105°C, 125°C, and 115°C, respectively (Figure 4E). This might be the result of the synergistic effect of heating temperature and time. The higher the temperature and the longer the time, the lower the content of ascorbic acid. Nonetheless, in comparison to boiling, frying, and microwaving methods, the steaming process preserved higher contents of ascorbic acid after cooking (38). Moreover, high hydrostatic pressure of 100–400 MPa treatment under 20–30°C retained a significantly higher content of ascorbic acid in vegetable beverage than traditional thermal treatment (39).

### 3.6 Volatile compounds of steamed sweet potato

**Figure 5:**
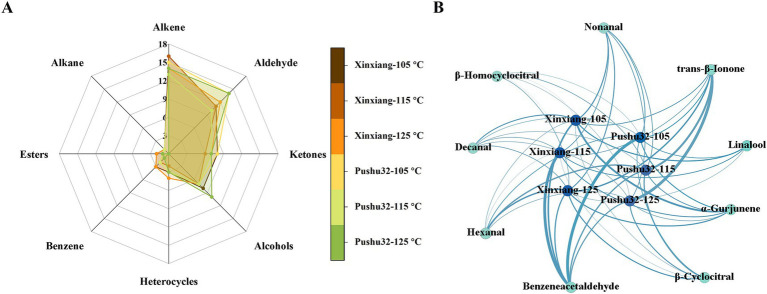
GC–MS analysis of volatile compounds in steamed sweet potato “Xinxiang” and “Pushu32” at different temperatures **(A)** Classes of volatile compounds. **(B)** Correlation network between different treatments and volatile compounds of steamed sweet potato. The inner nodes represent different treatments while the outer nodes represent volatile compounds. The thickness of the line is proportional to the content of volatile compounds. The color depth of the inner circle depends on the amounts of volatile compounds, while the color depth of the outer circle depends on the content of specific volatile components.

Steaming could better retain the food aroma (36). GC–MS was performed to examine the volatile compounds of high pressure steamed sweet potato (Table 2). 75 volatile components were identified in steamed sweet potatoes, including 20 alkenes, 15 aldehydes, 11 ketones, 19 alcohols, 4 heterocycles, 3 benzenes, 2 esters, and 1 alkane (Figure 5A). In agreement with our study, aldehydes were one of the major volatile organic compounds in purple-fleshed sweet potatoes (6). Among these components, the contents of *α*-Gurjunene and nonanal decreased with increasing temperature (Table 2). The hexanal was more accumulated in “Xinxiang” than “Pushu32”, while furfural was more accumulated in “Pushu32” than “Xinxiang” (Table 2). Based on the published research of chestnut-like aroma (40), the identified volatile compounds (Table 2) in sweet potato showed that nonanal, (E)-2-nonenal, and decanal might be the origin of chestnut-like aroma of “Xinxiang,” which presented higher contents than those of “Pushu32.”

**Table 2:** Volatile compounds of steamed sweet potato “Xinxiang” and “Pushu32” at different temperatures.

Class	No	Compound	Molecular	105°C		115°C		125°C	
				Xinxiang	Pushu32	Xinxiang	Pushu32	Xinxiang	Pushu32
Alkene	1	*α*-Terpinene	C_10_H_16_	1.34 ± 0.24	—	—	0.12 ± 0	1.00 ± 0	—
	2	*δ*-EIemene	C_15_H_24_	3.18 ± 0.22	0.61 ± 0	0.64 ± 0	0.80 ± 0.20	4.13 ± 0.22	—
	3	*α*-ionene	C_13_H_20_	2.84 ± 0.05	1.59 ± 0	1.02 ± 0.03	0.66 ± 0.17	3.51 ± 0	1.72 ± 0.10
	4	(+)-Cyclosativene	C_15_H_24_	5.27 ± 3.40	6.08 ± 1.74	4.02 ± 0.58	2.68 ± 0.34	10.26 ± 1.67	4.40 ± 1.87
	5	*α*-Copaene	C_15_H_24_	2.60 ± 1.60	1.42 ± 0.23	1.28 ± 0.67	0.76 ± 0.04	3.96 ± 1.23	1.41 ± 0.33
	6	*β*-Caryophyllene	C_15_H_24_	2.48 ± 0.90	—	—	—	—	—
	7	Cyperene	C_15_H_24_	6.88 ± 1.92	5.80 ± 2.01	5.84 ± 2.40	3.72 ± 0.53	13.71 ± 0.84	7.91 ± 0.89
	8	*β*-Copaene	C_15_H_24_	1.58 ± 1.00	—	—	0.21 ± 0	1.28 ± 0	—
	9	*γ*-Elemene	C_15_H_24_	3.04 ± 0.44	1.82 ± 0.08	1.18 ± 0.24	0.52 ± 0	3.85 ± 1.51	1.64 ± 0.33
	10	*α*-Guaiene	C_15_H_24_	2.21 ± 0.44	1.36 ± 0.34	0.80 ± 0.13	0.78 ± 0.43	2.56 ± 1.11	1.14 ± 0.08
	11	Guaia-6,9-diene	C_15_H_24_	1.48 ± 0.68	0.74 ± 0.34	0.26 ± 0	0.48 ± 0.42	1.06 ± 0.0	0.38 ± 0.08
	12	*α*-Humulene	C_15_H_24_	3.69 ± 1.12	1.50 ± 0.44	1.97 ± 1.12	0.90 ± 0.27	5.69 ± 1.17	1.31 ± 0.03
	13	*γ*-Muurolene	C_15_H_24_	2.19 ± 0	2.31 ± 1.25	1.83 ± 0.30	0.74 ± 0.19	3.85 ± 2.56	3.64 ± 0.28
	14	*α*-Muurolene	C_15_H_24_	1.29 ± 0	1.27 ± 0.42	0.28 ± 0	0.26 ± 0	—	1.09 ± 0.68
	15	*α*-Gurjunene	C_15_H_24_	24.02 ± 6.39a	10.35 ± 3.41b	7.72 ± 0.16ab	6.29 ± 3.09ab	27.20 ± 3.57b	7.38 ± 0.86b
	16	*δ*-Cadinene	C_15_H_24_	3.01 ± 1.94	0.80 ± 0.06	0.73 ± 0.48	0.55 ± 0.01	4.74 ± 0.78	0.76 ± 0
	17	Cyclohexene, 1,5,5-trimethyl-6-acetylmethyl-	C_10_H_16_O	—	1.02 ± 0.15	0.30 ± 0	0.22 ± 0	—	1.16 ± 0.08
	18	*β*-Elemene	C_15_H_24_	—	2.39 ± 0.38	0.59 ± 0	0.62 ± 0	—	3.36 ± 0.05
	19	*α*-Ylangene	C_15_H_24_	—	1.04 ± 0.34	0.34 ± 0	0.58 ± 0	—	—
	20	(+)-Aromadendrene	C_15_H_24_	1.14 ± 0.0	1.80 ± 0	1.13 ± 0.20	0.74 ± 0	1.17 ± 0	—
Aldehyde	1	Hexanal	C_6_H_12_O	4.93 ± 0.27	15.92 ± 3.75	9.65 ± 11.40	4.28 ± 0.01	5.35 ± 1.78	6.64 ± 0.38
	2	Furfural	C_5_H_4_O_2_	2.28 ± 0.32	5.93 ± 4.53	0.69 ± 0	3.41 ± 0.03	23.80 ± 25.59	17.28 ± 1.49
	3	Benzaldehyde	C_7_H_6_O	6.70 ± 0.75	6.03 ± 0.11	2.76 ± 0	2.55 ± 0.18	15.05 ± 4.85	7.00 ± 0.78
	4	Octanal	C_8_H_16_O	2.79 ± 0.70a	1.78 ± 0.38ab	1.14 ± 0.11ab	0.71 ± 0.39ab	3.18 ± 0.22b	1.89 ± 0.25ab
	5	Benzeneacetaldehyde	C_8_H_8_O	21.00 ± 4.47	15.26 ± 6.1	8.06 ± 9.01	17.80 ± 9.81	85.52 ± 60.88	15.89 ± 3.31
	6	Nonanal	C_9_H_18_O	14.94 ± 1.82a	8.72 ± 0.83b	6.71 ± 0.75ab	4.88 ± 0.45b	21.80 ± 1.06b	10.36 ± 2.25b
	7	(E)-2-Nonenal	C_9_H_16_O	6.85 ± 2.53	2.75 ± 0.74	3.01 ± 3.26	2.23 ± 1.18	13.1 ± 3.68	8.59 ± 0.61
	8	Decanal	C_10_H_20_O	12.80 ± 4.91	4.87 ± 0.06	3.97 ± 3.35	2.52 ± 0.91	14.66 ± 2.06	6.11 ± 1.19
	9	*β*-Cyclocitral	C_10_H_16_O	4.08 ± 1.41	14.63 ± 0.44	7.51 ± 7.39	4.12 ± 4.01	6.41 ± 2.79	14.86 ± 2.55
	10	*β*-Homocyclocitral	C_11_H_18_O	1.29 ± 0	7.07 ± 0.55	3.30 ± 0	1.59 ± 0	1.28 ± 0	4.47 ± 0.88
	11	(2E)-2-Methyl-4-(2,6,6-trimethyl-1-cyclohexen-1-yl)-2-butenal	C_14_H_22_O	0.49 ± 0	1.31 ± 0	0.83 ± 0.30	0.54 ± 0.61	0.95 ± 0.17	2.78 ± 0.08
	12	Heptanal	C_7_H_14_O	—	0.59 ± 0.02	0.54 ± 0.48	0.09 ± 0	0.33 ± 0	0.38 ± 0.10
	13	*α*-Cyclocitral	C_10_H_16_O	—	1.19 ± 0.28	0.82 ± 0	0.29 ± 0	—	0.58 ± 0.03
	14	Terpinen-4-ol	C_10_H_18_O	2.94 ± 0	2.10 ± 0.59	—	—	—	—
	15	(E)-2-Octenal	C_8_H_14_O	—	—	—	—	—	6.04 ± 0.30
Ketones	1	6-Methyl-5-hepten-2-one	C_8_H_14_O	0.56 ± 0	0.93 ± 0	0.67 ± 0	0.27 ± 0	1.17 ± 0	1.16 ± 0
	2	2,2,6-Trimethylcyclohexanone	C_9_H_16_O	2.70 ± 0.29	4.45 ± 0.74	3.13 ± 3.10	1.36 ± 0.99	3.01 ± 1.17	2.15 ± 0.40
	3	2-Undecanone	C_11_H_22_O	4.32 ± 0.68	1.57 ± 0.30	0.75 ± 0	1.50 ± 0.40	5.52 ± 0.61	1.69 ± 0.33
	4	1-(2,2-Dimethylcyclohexyl)ethanone	C_10_H_18_O	0.70 ± 0	—	—	—	—	—
	5	*β*-Damascenone	C_13_H_20_O	1.29 ± 0.05b	6.08 ± 1.74a	1.69 ± 0.26ab	2.15 ± 1.66ab	4.07 ± 2.40b	7.91 ± 0.83a
	6	Dihydropseudoionone	C_13_H_22_O	3.81 ± 2.82	2.92 ± 1.65	1.93 ± 1.23	1.20 ± 0.29	6.07 ± 2.56	5.03 ± 0.51
	7	trans-*β*-Ionone	C_13_H_20_O	9.91 ± 4.76c	23.24 ± 2.58ab	9.52 ± 2.56bc	5.52 ± 4.39c	16.50 ± 3.34c	39.14 ± 0.45a
	8	Ipomeamarone	C _15_H_22_O_4_	7.48 ± 6.53	—	—	0.69 ± 0	16.84 ± 3.85	3.61 ± 0
	9	Ionone	C_13_H_20_O	—	4.76 ± 0.04	1.29 ± 0	1.51 ± 0	—	4.22 ± 0.13
	10	Isophorone	C_9_H_14_O	—	2.63 ± 0	—	1.28 ± 0	—	—
	11	Pyranone	C_5_H_14_O_2_	—	—	—	1.41 ± 0	—	1.52 ± 0
Alcohols	1	Linalool	C_10_H_18_O	14.24 ± 6.73	—	2.11 ± 0	3.99 ± 0	22.58 ± 6.41	—
	2	Terpinen-4-ol	C_10_H_18_O	6.44 ± 0.75	0.85 ± 0	0.77 ± 0	0.66 ± 0	2.68 ± 0.0	—
	3	p-Cymen-8-ol	C_10_H_14_O	0.95 ± 0.51	—	—	—	—	—
	4	L-*α*-Terpineol	C_10_H_18_O	4.52 ± 1.34	1.61 ± 0.17	1.07 ± 0.88	2.04 ± 1.42	7.30 ± 2.34	2.15 ± 0.83
	5	2,4-Dimethylcyclohexanol	C_8_H_16_O	1.89 ± 0.02ab	2.90 ± 0.44a	1.22 ± 0.89ab	1.06 ± 0.31ab	2.34 ± 0.72b	2.93 ± 0.3ab
	6	Cubebol	C_15_H_26_O	2.23 ± 0.36	—	—	0.21 ± 0.0	1.23 ± 0	—
	7	2-cis-9-Octadecenyloxyethanol	C_20_H_38_O_2_	0.58 ± 0	—	—	—	—	—
	8	2-Benzofuranmethanol, 2,4,5,6,7,7a-hexahydro-4,4,7a-trimethyl-, cis-	C_11_H_18_O_2_	1.21 ± 0.44	—	0.68 ± 0	0.22 ± 0.0	2.06 ± 1.06	—
	9	1-Octen-3-ol	C_8_H_16_O	—	1.95 ± 0.28	0.85 ± 0	0.42 ± 0.0	—	—
	10	cis-*β*-Terpineol	C_10_H_18_O	—	5.42 ± 0.17	2.61 ± 0	1.47 ± 0.0	—	3.31 ± 0.03
	11	1-Methylcycloheptanol	C_8_H_16_O	—	2.37 ± 0.91	0.82 ± 0	0.58 ± 0.0	—	2.48 ± 0.03
	12	Citronellol	C_10_H_20_O	2.11 ± 0	—	—	—	2.51 ± 0	—
	13	2,6,6-Trimethylcyclohex-2-ene-1-methanol	C_10_H_18_O	—	—	—	—	—	—
	14	2-Furanmethanol	C_5_H_6_O_2_	—	—	—	0.51 ± 0	3.29 ± 0	0.83 ± 0
	15	(2E)-2-Methyl-4-(2,6,6-trimethyl-1-cyclohexen-1-yl)-2-buten-1-ol	C_11_H_18_O	—	0.36 ± 0	—	—	—	0.51 ± 0
	16	trans-Geranylgeraniol	C_20_H_36_O_2_	—	0.74 ± 0.23	—	0.15 ± 0	—	0.78 ± 0.18
	17	(4-Isopropyl-2-cyclohexen-1-yl)methanol, trans-	C_10_H_18_O	—	—	—	—	—	1.72 ± 0.10
	18	Epicubebol	C_15_H_26_O	—	—	—	—	—	0.83 ± 0.15
	19	(4,4,7a-Trimethyl-2,4,5,6,7,7a-hexahydro-1-benzofuran-2-yl)methanol	C_12_H_20_O_2_	—	—	—	—	—	2.68 ± 0.18
Heterocycles	1	Pyridine	C_5_H_5_N	3.33 ± 1.82	1.84 ± 0	1.10 ± 0	9.17 ± 12.15	19.73 ± 19.73	6.75 ± 0.58
	2	2-Pentylfuran	C_9_H_14_O	5.0 ± 1.17	6.73 ± 2.37	2.47 ± 0	2.04 ± 1.00	4.29 ± 0	3.59 ± 1.24
	3	1,4,6-Trimethyl-1,2,3,3a,4,7,8,8a-octahydro-4,7-ethanoazulene	C_15_H_24_	0.83 ± 0	1.59 ± 0.19	0.54 ± 0	0.17 ± 0	0.95 ± 0	3.26 ± 0.13
	4	Furan, 3-(4,8-dimethyl-3,7-nonadienyl)-, (E)-	C_13_H_20_O	—	—	—	—	1.56 ± 0	—
Benzene	1	p-Xylene	C_8_H_10_	2.02 ± 0.75	0.38 ± 0	0.41 ± 0.05	0.44 ± 0	0.61 ± 0	—
	2	Benzene, 1-methyl-3-(1-methylethyl)-	C_9_H_12_	1.85 ± 0.29	—	—	0.19 ± 0	1.11 ± 0	—
	3	2-Methoxy-4-vinylphenol	C_9_H_10_O_2_	1.80 ± 0.90bc	2.18 ± 0.17bc	0.80 ± 0.14bc	1.25 ± 0.04b	3.23 ± 0.22c	6.11 ± 0.66a
Esters	1	Dihydroactinidiolide	C_11_H_16_O_2_	1.53 ± 0.24bc	2.20 ± 0.15b	1.26 ± 0.01bc	0.64 ± 0.42bc	2.73 ± 1.00c	4.88 ± 0.66a
	2	Oxime-, methoxy-phenyl-_	C_8_H_9_NO_2_	1.09 ± 0	—	—	—	2.23 ± 0	—
Alkane	1	n-Tetradecane	C_14_H_30_	1.72 ± 0	—	0.36 ± 0	0.43 ± 0	3.51 ± 1.67	—

The contents of volatile compounds were expressed in nmol·L^−1^. Different superscript letters in the same line represent significant differences among groups (*p* < 0.05).

According to the sensory evaluation results, the aroma of steamed sweet potatoes at 105°C (120.8 kPa) was the best, so the 9 aroma components (trans-*β*-ionone, hexanal, benzeneacetaldehyde, *β*-cyclocitral, *α*-gurjunene, nonanal, linalool, decanal, *β*-homocyclocitral) with the highest contents at 105°C are further analyzed. Correlation network between different treatments and volatile compounds of steamed sweet potato was performed and shown in Figure 5B. Benzeneacetaldehyde accounting for floral and sweet attributes in aroma (41) was one of the characteristic aroma substances, more accumulated in “Xinxiang115” (169.1 kPa) and “Pushu32105” (120.8 kPa) treatments (Figure 5B). Benzeneacetaldehyde possessed an extremely high flavor dilution factor of 512, thus can be detected at a very low concentration. Metabolic profiling revealed the potential essential volatile component in shoots and roots of tea plants including trans-*β*-Ionone, a floral and fruity compound (42). In our results, a higher content of trans-*β*-Ionone was found in “Pushu32125” (232.1 kPa). *α*-Gurjunene with a spicy and woody aroma was detected to accumulate more in “Xinxiang105” (120.8 kPa), and was often applied as food additives and perfumes to enhance fresh-smelling fragrance. Also, *α*-Gurjunene contributed to the wood and balsamic flavors of Bobaizhi (43). Moreover, nonanal was the degradation product of palmitoleic acid, and could be used as an aroma enhancer in food (44). Consistent with our study, nonanal was also one of the pivotal aroma compounds of steamed green tea (45). Identifying the volatile composition of sweet potato offered a theoretical basis to reveal the formation mechanism of aroma and further improve the processing quality of sweet potato.

### 3.7 Association analysis between quality and aroma of steamed sweet potato

**Figure 6:**
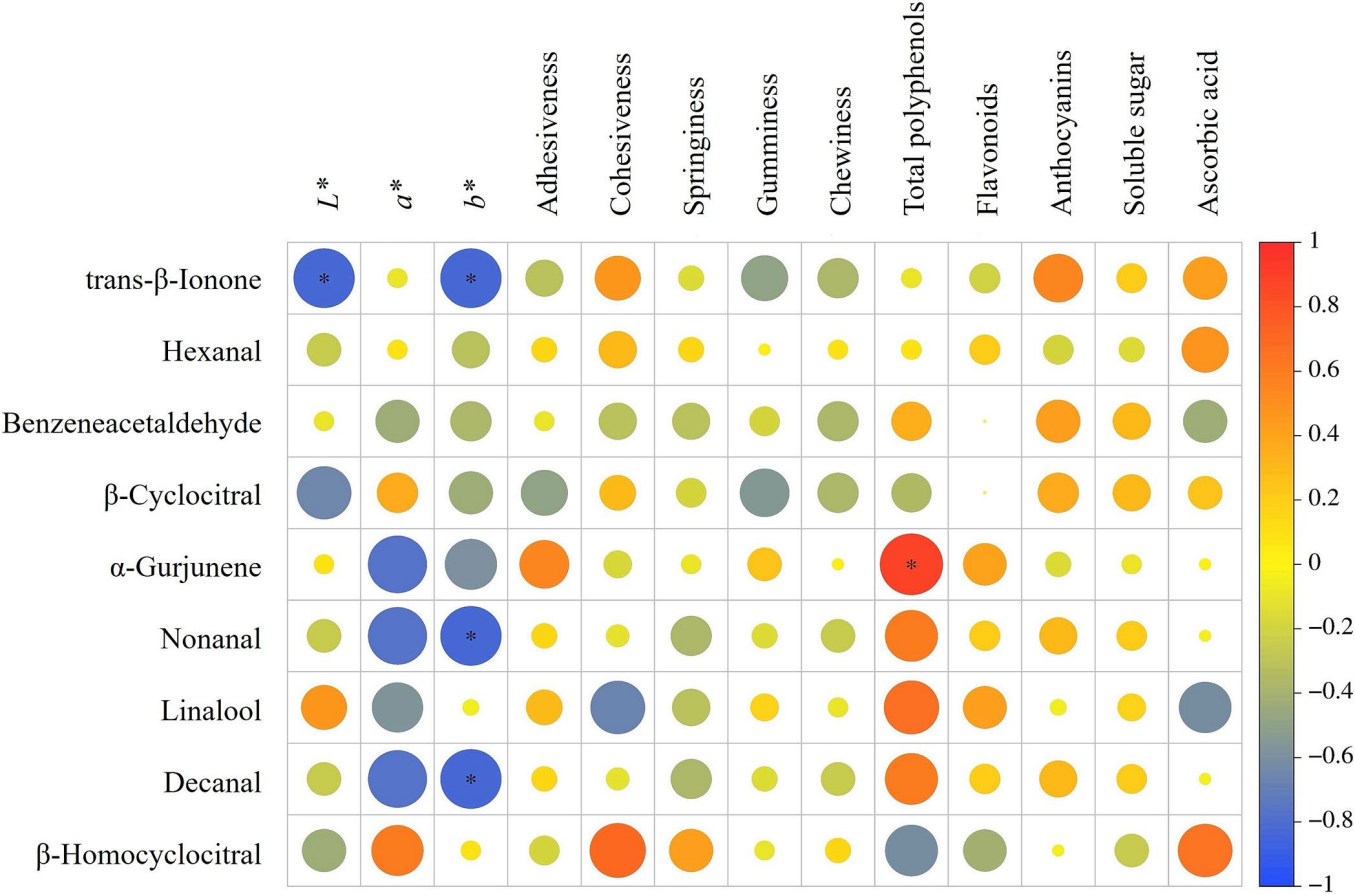
Spearman correlation analysis between qualities and aroma of steamed sweet potato The color intensity (red and blue) and the circle size indicate the correlation strength. “*” represents *p* < 0.05.

Based on the correlation analysis of Figure 6, trans-*β*-ionone content was significantly negatively correlated with L* of steamed sweet potato chunks. The trans-*β*-ionone, nonanal, and decanal contents were significantly negatively correlated with b* of steamed sweet potato. These results indicated that the increase of trans-*β*-ionone, nonanal, and decanal aroma contents might lead to dull color of sweet potato. Besides, the *α*-gurjunene content was significantly positively correlated with total polyphenols of steamed sweet potato, suggesting that the presence of polyphenol might be conducive to the production of *α*-gurjunene, and affects the wood aroma.

## 4 Conclusion

Steaming is a convenient and healthy cooking method widely used in households and industry, to heat the food evenly and retain its original taste and nutrition. Here, the impacts of different steaming temperatures (105°C, 115°C, and 125°C) × pressures (120.8 kPa, 169.1 kPa, and 232.1 kPa) × time (35, 25, and 15 min) on the nutritional value, phenolic compounds, water status, volatile compounds, and sensory properties of sweet potato “Xinxiang” and “Pushu32” were investigated. In comparison to the soft “Pushu32”, the chewy “Xinxiang” had a significantly lower fiber score and exhibited greater changes of adhesiveness, cohesiveness, chewiness, and gumminess after steaming. According to the sensory evaluation, the optimal steaming conditions were 125°C (232.1 kPa) × 15 min for “Xinxiang,” and 115°C (169.1 kPa) × 25 min for “Pushu32”. The low-field nuclear magnetic resonance indicated that more than 90% of water in sweet potato was immobilized water, and 125°C high pressure steaming enhanced the bound water proportion, which might contribute to the adhesive texture. 125°C × 15 min treatment showed higher contents of anthocyanin, soluble sugar and lower content of ascorbic acid in sweet potato. Moreover, 75 volatile components were identified in steamed sweet potato using GC–MS. Correlation network analysis implied that benzeneacetaldehyde, trans-*β*-ionone, *α*-gurjunene, and nonanal were pivotal aroma compounds of steamed sweet potato. The trans-*β*-ionone, nonanal, and decanal aroma contents might be correlated with dull color of sweet potato. The presence of polyphenols might be conducive to the production of *α*-gurjunene, and affects the wood aroma. Based on all the indicators, the active components of two varieties of sweet potatoes were better retained by steaming at 125°C. In a word, our research provided new insights into the sensory quality formation mechanism and steaming process optimization.

## Data Availability

The original contributions presented in the study are included in the article/supplementary material, further inquiries can be directed to the corresponding authors.
